# Reexamining the use of race in medical algorithms: the maternal health calculator debate

**DOI:** 10.3389/fpubh.2024.1417429

**Published:** 2024-06-13

**Authors:** Rachel Wangari Kimani

**Affiliations:** Laboratory of the Neurogenetics of Language, Rockefeller University, New York, NY, United States

**Keywords:** health disparities, VBAC, race, clinical algorithms, equity, maternal mortality

## Abstract

The concept of race is prevalent in medical, nursing, and public health literature. Clinicians often incorporate race into diagnostics, prognostic tools, and treatment guidelines. An example is the recently heavily debated use of race and ethnicity in the Vaginal Birth After Cesarean (VBAC) calculator. In this case, the critics argued that the use of race in this calculator implied that race confers immutable characteristics that affect the ability of women to give birth vaginally after a c-section. This debate is co-occurring as research continues to highlight the racial disparities in health outcomes, such as high maternal mortality among Black women compared to other racial groups in the United States. As the healthcare system contemplates the necessity of utilizing race—a social and political construct, to monitor health outcomes, it has sparked more questions about incorporating race into clinical algorithms, including pulmonary tests, kidney function tests, pharmacotherapies, and genetic testing. This paper critically examines the argument against the race-based Vaginal Birth After Cesarean (VBAC) calculator, shedding light on its implications. Moreover, it delves into the detrimental effects of normalizing race as a biological variable, which hinders progress in improving health outcomes and equity.

## 1 Introduction

The debate on racial categorization in healthcare persists, challenging the long-standing integration of race in medical diagnostics and treatment against the backdrop of social scientists’ consensus that race holds no substantial biological foundation (1, 2). This divergence is starkly illustrated by the persisting health disparities across racial lines, with the alarming maternal mortality rates among African-American women—3.55 times higher than those of their White counterparts between 2016 and 2017—standing as a testament to systemic inequities (3). One emblematic medical algorithm case is the race-based Vaginal Birth After Cesarean (VBAC) calculator. This tool’s potential biases may exacerbate health disparities by using race as a determinant in clinical decision-making, thereby reducing the likelihood that Black and Hispanic women will be recommended for Trial of Labor After Cesarean (TOLAC).

Medical algorithms are tools used to systematically approach clinical problems or treatment paths to reduce errors and improve healthcare (4). The VBAC calculator assesses the likelihood of a successful trial of labor after cesarean (TOLAC), factoring in BMI, maternal age, and previous cesarean details (5). However, its inclusion of race/ethnicity, despite aiming to refine predictions, risks reinforcing racial biases by suggesting unsubstantiated biological differences and, thus, potentially different care standards. However, its inclusion of race/ethnicity, despite aiming to refine predictions, risks reinforcing racial biases by suggesting unsubstantiated biological differences. Associating race—a social construct—with innate biological capabilities, such as the ability to give birth vaginally after a cesarean section, not only perpetuates stereotypes but also potentially guides clinicians toward different standards of care based on race. This raises significant concerns about fairness and equity in medical practices. The debate surrounding the VBAC calculator highlights the challenge of leveraging technology to improve healthcare outcomes without perpetuating societal biases (6–8). Medical algorithms must be transparent, unbiased, and inclusive, minimizing past biases and prioritizing individual clinical characteristics to improve healthcare delivery (9).

This paper examines the push to phase out the race-based VBAC calculator, positioning this initiative within the broader discourse surrounding the intersections of technology, artificial intelligence, social justice, and the pursuit of equity in healthcare. It confronts the detrimental implications of conflating race with biological differences—a legacy rooted in the era of slavery and the historic exploitation of Black, Indigenous, and People of Color (BIPOC) in the development of science and medicine. Persisting in this practice not only deepens discriminatory patterns but also hinders progress in removing racism as a determinant of health outcomes (Jones, 2021). This exploration traces the origins of racial classification in American science and medicine, evaluates the debates surrounding the VBAC calculator, and critically examines the scientific and ethical underpinnings of race-based medical algorithms. This analysis advocates redefining race as a socio-political, rather than biological, category to foster a more equitable and just healthcare system.

## 2 History of racial categories in science and medicine

The idea of racialization (classifying people by race) appeared in English in the 1500s (10). In the 1700s, Carl Linnaeus, a taxonomist famous for categorizing plants and animals, proposed four classifications of humans based on skin color: Europaeus (white skin), Americanus (reddish), Asiaticus (tawny/tan), and Africanus (blackish) (11). Later, Blumenbach, a student of Linnaeus, divided humans into five groups based on geography and physical characteristics. In his classification, Caucasians were light-skinned people from Europe, and people living near Asia and Africa proximal to Europe were (Mongolians), Ethiopians (dark-skinned Africans), Americans (New World natives), and Malays (Polynesians) (12). Both Linnaeus and Blumenbach assumed a scientific stance, but their bias toward the social superiority of their European ancestry was evident in their writings. This presumption evolved into an established hierarchical order in which Europeans were at the top and Africans at the bottom, a moral justification for slavery, colonization, genocide, and discriminatory laws such as Jim Crow laws (13).

The concept of race was both politically and scientifically pivotal as the demographic composition of the United States evolved with the arrival of more immigrants. The first naturalization law in the United States, passed in 1790, restricted citizenship to “free white persons,” thus institutionalizing racial categorization (14). Throughout history, the U.S. Census has played a significant role in shaping and reflecting categories of race. Initially, census enumerators identified individuals’ race based on their perception, using categories such as “free White persons, enslaved people, or all other free persons” (15). As immigration and societal views on race changed, so did the categories, expanding to include mixed-race identifications such as mulatto, quadroon, and octoroon, and later detailed listings for Asian and Hispanic groups (15).

A significant change occurred in 1970 when the Census transitioned from enumerator identification to self-identification. This shift marked a crucial change in the control over racial identity as it allowed individuals to define their own racial identity. As a result of this methodological change, there were significant increases in the counts of some groups, particularly the American indigenous population (16). This shift emphasizes the fluidity of racial categories and highlights how they are influenced by social and political constructs rather than immutable biological differences.

The evolution of race statistics illuminates the utilization of physical appearances for categorizing individuals and as tools for political and social control. This reflects the broader dynamics of power and colonization that have shaped racial identities in America. The term “Indian” was initially used to “other” and marginalize the diverse Indigenous populations of America, further facilitating their exclusion from the nation-building process (15). As Irish immigrants and later other groups gradually assimilated into American culture, their racial categorization shifted, affording them the political and economic privileges reserved for Whites (17). This manipulation of racial categories to “other” various groups demonstrates how race was wielded as a tool for political and social control, showcasing the complex interplay of race, power, and identity in American history.

In science, the concept of race has been intertwined with ideologies of white supremacy, fueling movements that have led to the discrimination, elimination, and mistreatment of people of color under the guise of scientific advancement (18–20). Politicians such as Theodore Roosevelt and Winston Churchill supported the hypothesis of societal improvement using eugenics, a form of racial science focused on selective breeding and controlling human reproduction to achieve desired genetic traits (21). This highlights how genetics, as a scientific discipline, is an example of an area founded on racial ideology.

The renowned geneticist Francis Galton played a pivotal role in this history when he conceptualized eugenics in 1883. He described a range of physical, mental, and moral traits across races, arguing for the selective propagation of traits associated with the White race to improve societal health (22). This ideology was not isolated to genetics but extended into other scientific areas such as statistics. Figures like R.A. Fisher and Karl Pearson, credited with developing modern statistical methods, were deeply entrenched in the eugenics movement in England (23). They supported policies like sterilization of those with mental disabilities and race-based immigration controls.

In 1923, Henry F. Osborn, the then-president of the American Museum of Natural History in New York, publicly called upon the government to recognize the biological racial differences and preserve the virtue of the White race. Eugenics scientists received financial support from private donors, associations such as the American Breeders Association, and the government. The Eugenic Records Office in the Carnegie Institution was established by a prominent evolution scientist, Charles Davenport, who recruited Harry H. Laughlin as the superintendent of the ERO. The main goal of the ERO was to gather data supporting the eugenic movement and educate the public on the importance and implications of eugenic research. Laughlin was appointed as a congressional expert eugenics agent by the US Congress Committee on Immigration. In 1922, he published a book on eugenic sterilization in the United States, arguing against integrating races and sterilizing individuals with mental disability (18). This contributed to state laws legalizing the sterilization of persons living with cognitive disabilities and later led to the mass forced sterilization of Indigenous and Black people in the United States (24).

These historical instances are a stark reminder of the enduring impact of race-based ideologies on shaping scientific thought and practice. By the early 20th century, the eugenics movement had grown into a significant scientific movement in the early 20th century, with American and European scientists embracing racial ideology as a science. They conducted experiments to propagate these false narratives and taught these concepts in universities, conferences, and even publicly (21). The legacy of these actions continues to influence the scientific landscape today, highlighting the crucial need for ongoing scrutiny and reform in how racial concepts are integrated into scientific research and discourse.

The embedding of eugenics in science created a significant challenge for cultural anthropologists and intellectuals, including W.E.B. De Bois and Franz Boas, who sought to counter the illogical racist theories. Franz Boas, widely regarded as a founder of modern cultural anthropology, employed scientific reasoning to refute earlier claims that Black people have smaller brains. For instance, he conducted a meticulous study measuring human skulls to provide evidence contradicting these assertions (25). On the other hand, Du Bois approached the issue of race from a social and historical perspective, viewing it as a mechanism used to group individuals and actively perpetuate economic and political oppression.

Eugenic policies were formally purged after World War II after the Nazi eugenics atrocities and the United Nations declared that race is a social construct (26). Though the United States denounced racial science officially, the ideology was already embedded into the power structures, particularly in science and immigration laws that favored White persons’ immigration and discouraged interracial marriages. American publications of the American Eugenics Association and American Genetics Association fueled most Nazi eugenics atrocities (21). It is, therefore, no surprise that the idea of race science continues in scientific discourse and medical application of emerging genetic technologies that attempt to assign social and medical outcomes to immutable racial differences.

## 3 Evolution of racial categories in modern medicine

The early 2000s saw a revolutionary development in human genome sequencing, enabling a comprehensive insight into an individual’s genetic makeup, encompassing variations, mutations, and potential disease markers within their DNA. Despite the scientific evidence, genetic similarities often surpass differences among individuals from different racial groups (11, 27). Furthermore, the persistence of racial biological essentialism, characterized by the belief in race as a biological and genetic entity, continues to have significant social and political ramifications. This enduring notion, despite its scientific debunking, underscores how deeply ingrained and complex the issue of race remains in our society, influencing both societal interactions and governmental choices.

**Figure 1:**
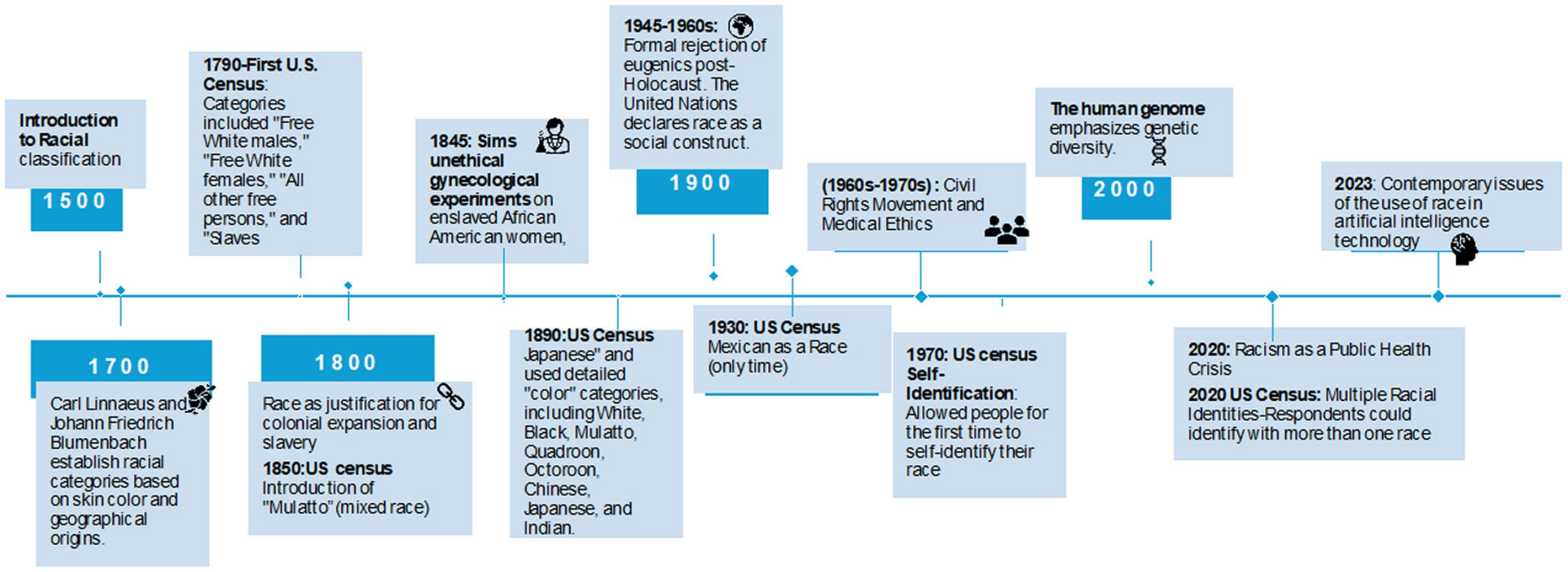
Chronology of racial categorization and its impact in medicine and society. The timeline represents a selection of key events that have influenced the concept and application of racial categorization in medicine and its broader societal implications. It is not exhaustive but highlights pivotal moments shaping current perspectives on race in medical practices and technologies. The historical “color” categories used in the U.S. Census reflect the sociopolitical constructs of race during specific periods and may be considered outdated and insensitive today. “White” referred to individuals of European descent; “Black” to those of African descent; “Mulatto” indicated mixed African and European ancestry; “Quadroon” and “Octoroon” described individuals with one-quarter or one-eighth African ancestry, respectively. “Chinese,” “Japanese,” and “Indian” referred to individuals from those respective ethnic or national origin.

An illustrative example is the US Census and Vital Statistics, which collects racial data corresponding to ancestry or geographical region- an adaptation of Blumenbach’s racial classification (see Figure 1). Since the 1980s, the US has also measured “Hispanic” as an ethnic group of people who speak Spanish and originate from Mexico, Puerto Rico, Cuba, Central and South America, and other Spanish-speaking countries except for Portugal and Brazil (13). Definitions of race and ethnicity have evolved to reflect cultural and social norms (13). Ethnicity, which may overlap with race, is a subjective label for people who share cultural, language, or physical attributes (11). Similar to race, ethnicity is a social construct with complex and fluid dimensions that are difficult to measure scientifically. So, in the case of VBAC, how did race (being “Black”) and ethnicity (being “Hispanic’) become negative variables in a clinical tool?

## 4 Case study: the VBAC calculator and maternal health

Cesarean delivery, an abdominal surgery for childbirth, carries various risks that can affect both mother and child, potentially leading to increased rates of mortality and morbidity (28). Over the past few decades, cesarean rates in the United States have surged, reaching 32% by 2009 (28). Traditionally, women who had undergone a cesarean were often expected to repeat the procedure for future births. This changed in the mid-1980s, as evidence emerged suggesting that Vaginal Birth After Cesarean (VBAC) could be a safe alternative for certain patients, offering reduced risks associated with repeat cesareans, thus prompting a shift toward encouraging Trial of Labor After Cesarean (TOLAC) (29).

In 2007, the Maternal-Fetal Medicine Units (MFMU), supported by the National Institutes of Health, created a VBAC calculator to aid healthcare providers in evaluating the viability of TOLAC for individual patients. This tool, which received endorsement from the American College of Obstetrics and Gynecologists, considers several factors, including body mass index (BMI), patient age, cesarean history, and race/ethnicity, to predict the success of VBAC (6, 30).

The VBAC calculator decreased the likelihood of VBAC success for women identified as African American/Black and Hispanic (Table 1). Using the assumption that Black and Hispanic women have less successful VBAC, the calculator subtracted from the score, which gave these women less chance of TOLAC. Consequently, women with the same age, BMI, and history of cesarean had different scores based on their identified race/ethnicity. Vyas et al. (6) challenged the VBAC’s use of these race-based correction factors. They argued that if scores influenced clinicians’ decisions, the calculator probably contributed to maternal disparities (6). Vyas (31) also noted that many other factors, such as marital status and insurance, were identified in creating and validating the tool that could have been incorporated into the predictive tool used in the United States, Israel, Italy, United Kingdom, Netherlands, and New Zealand (32). In fact, the version of the tool used in most countries does not include race correction. Therefore, embedding race/ethnicity corrections in the US-based VBAC creates inequitable treatment by race and further propagates the notion that racial disparities are immutable.

**Table 1:** Comparison of VBAC success rate calculations with and without race/ethnicity.

Input criteria	With race and ethnicity	Without race/ethnicity
Patient’s age	✓	✓
BMI	✓	✓
Previous cesarean	✓	✓
Race/ethnicity	✓	X
Sample predicted VBAC score	48.2% success rate (95% confidence interval 44.5, 51.9%)	64.5% success rate (95% confidence interval 62.1, 66.9%)

The sample VBAC success rates above were calculated for a hypothetical patient profile with the following characteristics: Maternal Age of 31 years, BMI of 22, no prior vaginal birth, and a previous cesarean section due to arrest of dilation. The rate for the African American/Hispanic column incorporates race/ethnicity as a risk factor, as per the original VBAC algorithm, whereas the rate for the Non-African American/Hispanic column excludes this variable, reflecting the updated calculation approach. The confidence intervals provide a statistical estimate of the range in which the actual success rate may fall.

The Maternal-Fetal Medicine Units Network (MFMU), which developed the race-adjusted VBAC calculator, intended to aid in clinical decision-making rather than to perpetuate discrimination against Black and Hispanic women. Nonetheless, the calculator’s race adjustments resulted in lower estimated VBAC success probabilities—by 5–15 percentage points—for Black and Hispanic patients compared to White patients with similar clinical profiles, based on analyses from a large cohort study with an evidence level II (30). Furthermore, their model suggested that patients with scores below 60 percent might reduce morbidity by opting for a repeat cesarean over attempting a VBAC (33). However, this calculator’s utilization of race as a biological variable is emblematic of a broader trend within epidemiological research. Historically, race and ethnicity have been employed as imprecise surrogates for complex social and health factors, thereby perpetuating a systemic issue where the scientific application of race may conceal actual social determinants of health. For example, factors such as differential access to healthcare, environmental exposures, and socio-economic disparities are critical but are often masked by the simplistic categorization by race (34). This reductionist approach can lead to misdiagnoses and inequitable health outcomes, as it overlooks the multifaceted nature of health determinants, such as the impact of living in high-pollution areas or the chronic stress associated with racial and economic marginalization (35).

The subsequent racial discrimination controversy surrounding the VBAC calculator prompted a reevaluation, leading Grobman and colleagues to revise the tool, replacing race/ethnicity with medical history components such as hypertension (5). This incident has sparked broader discourse on the critical need to reassess the role of race in clinical algorithms and to acknowledge the potential biases that arise from its misuse (31, 36).

## 5 Legacy of the VBAC calculator

The history of science and technology, such as genetics, shows how ideology influences science. In general, it is assumed that there is a separate scientific meaning of race in science that is not contaminated by the sociopolitical meaning of race. Roberts (1) argues that the biologization of race (use of race as an inherent biological fact) is acceptable today because racism is normalized, making it invisible. In medicine, there are existing race-based guidelines taught to clinicians currently in use. In a recent article, Amutah et al. (37) presented a case of a patient of mixed parental ancestry being considered for a kidney transplant. Given the existing race correction adjustment for Black patients in the glomerular filtration rate (GFR), the patient has differential access to the transplant list depending on which race he is considered to be.

In this kidney function test scenario, the racial differences come from a racist presumption that Black patients have greater muscle mass than other races; therefore, the GFR needs to be adjusted (38). These beliefs of Black individuals having denser muscle and thicker skin have been used to justify harmful medical practices. For example, in the 1950s, Black patients were dosed with higher X-ray radiations based on unsubstantiated beliefs grounded on racism (39). In this case, White individuals are assumed to be “normal,” and Black individuals or people of color need more X-rays to penetrate their skin. Similarly, in pain medicine, there has been an assumption that Black people have thick skin and feel less pain. This has led to the mistreatment of Black patients in medicine, including performing surgery without anesthesia.

In obstetrics, studies conducted in the 1920s relied on the racialized anatomy concepts published and propagated notions that White women had a standard pelvis ideal for childbirth. In contrast, Black and Indigenous women were assumed to be anatomically deficient (19). These assumptions of faulty anatomy led to high rates of interventions such as cesareans among women of color to compensate for their abnormalities (40). Additionally, these notions of the inferiority of Black and Indigenous women were used to justify forced sterilization (41). Despite the obvious racist antecedents of the inferiority of women of color pelvic anatomy, researchers continue to cite racial and ethnic variation in pelvic as a factor contributing to adverse childbirth outcomes (6).

## 6 Policy implications and future directions

A recent draft guideline from the National Institutes of Health and Care Excellence (NICE) suggests that labor induction should be considered at 39 weeks for women of Black, Asian, or minoritized background, even if the pregnancy is uncomplicated (42). The guideline recommends labor induction for White women at 41 weeks of gestation. Clinicians who argued against the recommendation noted that race has been used as a proxy for social and medical factors (43). Again, in this case, there is an implicit presumption that race confers immutable characteristics.

It is not scientifically accurate to use broad racial categories like Asian or African in clinical decisions. For instance, people categorized as Asians comprise a diverse group of individuals, including Chinese, Japanese, Indians, Filipinos, Thais, and others. This is a large geographical region with various social and cultural factors that impact health. Additionally, DNA sequencing has revealed significant variability within African populations. Thus, grouping Africans into one category does not make any biological sense. Race and ethnicity are fluid social constructs and unreliable indicators of ancestry or genetics.

The examples of VBAC and NICE guidelines’ scrutiny of the use of race illustrate a critical need to reexamine the institutionalization of racism in medicine. A recent systematic analysis of UpToDate articles showed that for articles that mention race, biologization of race occurred in 93.3% of the articles, and there were discussions of inherent racial differences without context (44). Furthermore, 32.7% of the articles racialized biomedical research and clinical practice. This included references to racialized patterns of behavior and cultural practices. There was also insufficient data on Black populations, limiting the study to a specific racial group and race-based clinical practice guidelines. The widespread use of these articles in clinical decision-making among clinicians and medical and nursing students raises the question of whether the normalization of race can be systematically dismantled.

In a recent article in Pediatrics, Wright et al. (45) argued that evidence from the human genome project, stress, and adaptation studies provide enough evidence to dismantle race-based medicine. Similarly, other researchers and clinicians have concluded that race is inaccurate in understanding human diversity and clinical race-based predictions (31). However, as Vyas et al. (31) explained, a lack of evidence of genetic races has not stopped the belief from manifesting insidiously in clinical practice. This belief is also true in the American general public. For example, a recent poll showed that differences in the socioeconomic status of White and Black people were due to genetics (27). These essentialist theories, especially among White individuals, reduce the support for policies that attempt to dismantle systemic racism- a social determinant of health.

Nonetheless, only a few systematic solutions are proposed apart from the slow progress of undoing race corrections one at a time. Kane et al. (36) proposed that clinicians and researchers use structurally just algorithms prioritizing social drivers of inequities such as insurance status, education, and economics. This alternative approach emphasizes structural justice by analyzing the root causes and working collaboratively with advocates and communities to address societal-level circumstances contributing to disparities such as those noted in maternal and child mortality. In addition, it may be prudent to return to the drawing board and decenter diagnostics, prognostic tools, and treatment guidelines from one racial group and instead create an inclusive approach to biomedical research and healthcare.

## 7 Conclusion

The history of science and technology shows that social ideologies influence science. Therefore, contrary to the prevalent use of race as a biological variable, evidence shows that genetic and biological races do not exist. The acceptability of race in medicine is particularly troubling since it has caused iatrogenic harm and possibly exacerbates health disparities. In the case of VBAC, creating medical algorithms that discriminate against Black and Hispanic women based on race and ethnicity (social constructs) detracts from efforts to improve maternal mortality. Further, using race causes harm by miscategorizing people based on fixed ideas of race, reinforces biological essentialism, and prevents support for reforms to eliminate racism as a social determinant of health.

## Data availability statement

The original contributions presented in the study are included in the article/supplementary material, further inquiries can be directed to the corresponding author.

## Author contributions

RK: Conceptualization, Formal analysis, Methodology, Visualization, Writing – original draft, Writing – review & editing.
